# Piloting Simulations: A Systematic Refinement Strategy

**DOI:** 10.7759/cureus.6434

**Published:** 2019-12-20

**Authors:** Celina Da Silva, Adam Dubrowski

**Affiliations:** 1 Medical Education and Simulation, York University, Toronto, CAN; 2 Health Sciences, Ontario Tech University, Oshawa, CAN

**Keywords:** refinement, simulation, simulation design scale, medical research council, modeling

## Abstract

Introduction

Few approaches articulate a systematic way to address confusing, missing, or underdeveloped simulation design features prior to implementing into coursework. To address this gap, we tested a novel, systematic refinement strategy to improve the design elements of two simulations.

Methods

Forty eligible participants (Year 3 undergraduate nursing students) evaluated two simulation scenarios (each followed by a debriefing session) through a novel and systematic refinement strategy across five iterations. Each simulation was evaluated using the validated Simulation Design Survey (SDS). Ratings were analyzed using descriptive data. Students also responded to an open-ended question in order to provide qualitative feedback regarding how to improve its features, i.e., scenario design and debriefing components. Written comments by students were analyzed using the principles of qualitative content analysis.

Results

Descriptive statistics revealed a gradual increase in the mean scores of the SDS over each of the simulation refinement periods. For the first simulation, the SDS mean score reached a high on Day 5 of 4.86 (standard deviation (SD) = 0.14) in contrast to a score of 3.45 (SD = 0.17) on Day 1. For the second simulation, the SDS mean score was 4.75 (SD = 0.16) on Day 5, which represented a mean score increase of 1.01 from the score on Day 1.

Conclusions

This novel refinement strategy improved the overall design elements of each of the simulations. The potential use of the SDS and open-ended feedback, guided by a refinement approach, merits further investigation.

## Introduction

Simulation-based learning (SBL) is a pedagogical approach that has innovated education for health professions. Simulation for educational purposes classically seeks to reproduce features of a real-world phenomenon with the aim of meeting learner outcomes [1]. Simulations can be designed in a variety of ways using high technology manikins and/or simulated persons and can be implemented via the internet or face-to-face [2-3]. While the evaluation of a simulation’s design features is a necessary component to ensure it runs as intended, the refinement phase remains a black-box. Few approaches articulate a replicable way to address confusing, missing, or underdeveloped simulation design elements prior to inclusion in the curriculum and with the intended learners.

Different methods are used to evaluate simulations in nursing education, such as questionnaires (i.e., the Simulation Design Scale (SDS)) and open-ended feedback/interviews. Researchers have conducted pilot studies [4-5] and pre- and post-tests with selected students [6] to evaluate the simulation design features as a measure of quality assurance in nursing programs. Although questionnaires, such as the SDS, were used to assess the simulation’s design features in the aforementioned studies, it is unclear whether the simulations underwent any refinement prior to the studies.

Several conceptual frameworks articulate how to develop simulations. The National League for Nursing (NLN) Jeffries simulation theory [7] is used in nursing education to guide the selection of a simulation’s learning objectives and the design of the simulation scenarios, including debriefing. Another is the scenario design framework by the Royal College of Physicians and Surgeons of Canada. It is a six-step guide used in the development of simulations: (a) identifying objectives and the educational strategy, (b) creating a scenario summary and clinical vignette, (c) selecting equipment, moulaging techniques, and adjuncts, (d) creating the scenario framework and stages, (e) programming the scenario, and (f) refining the simulations. However, conceptual approaches, such as the latter, do not articulate an explicit and systematic way to test the simulations to ensure its design is of high quality prior to implementing with learners or prior to inclusion as educational interventions in research studies. Other instructional design strategies to guide simulation development include the use of content experts, best practice guidelines, and reference to accreditation body standards of practice, such as the International Nursing Association for Clinical Simulation and Learning (INACSL). The INACSL best-practice standards highlight the importance of testing simulations prior to implementation with the learner but do not articulate a systematic way to conduct testing to optimize the quality of the simulations [8].

In this Phase 1 study, we propose a novel and systematic five iteration refinement algorithm with decision rules to test and optimize the design features of two simulations (each followed by a debriefing session) using the SDS and an open-ended question. The purpose was to evaluate the effectiveness of the refinement strategy applied to two separate simulations (each followed by a debriefing session). The study objectives were (a) to evaluate the scores of the SDS over two separate refinement periods using a systematic algorithm and decision rules, (b) to examine the categories identified by the participant's responses to the open-ended question, appended to the SDS, and (c) to examine the final timing of the two simulations (each followed by a debriefing session).

The Medical Research Council (MRC) framework was used as the conceptual framework to develop and assess how the design elements of each of the simulations related to and interacted with each other [9-11]. The MRC updated guidelines recommend an iterative, cyclical phased approach to intervention development and evaluation; moreover, it should emphasize that the neglect of adequate development, testing, and consideration of the practical issues of implementation will result in weaker interventions that are difficult to evaluate and less likely to be implemented. Concern for implementation should address Phase 1 in collaboration with stakeholders and their involvement in the design and feasibility processes.

## Materials and methods

**Figure 1:**
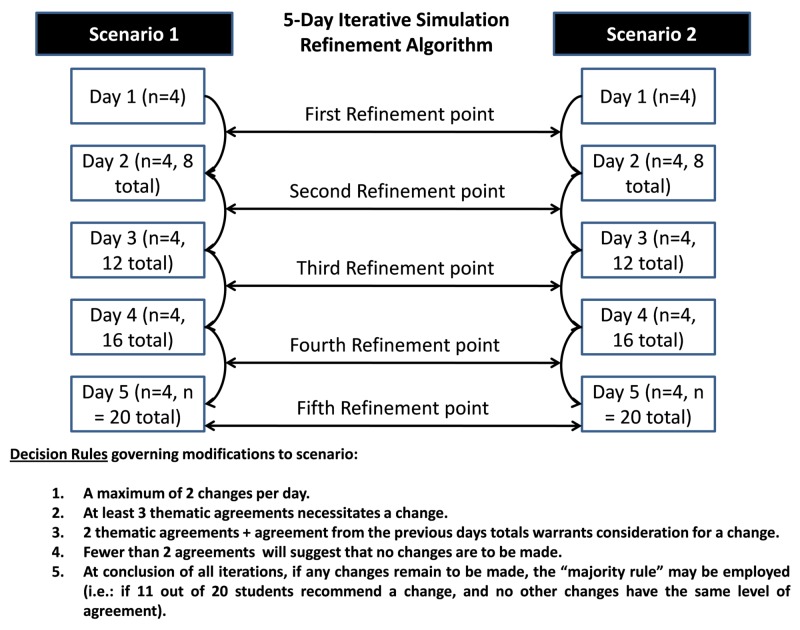
Refinement Strategy

Two refinement sessions were undertaken for the two simulation scenarios, each followed by a debriefing session. The simulations were tested and refined through the participation of four nursing student participants per day, per scenario, from Monday to Friday. Each successive day was an iteration: Monday was Iteration 1, Tuesday was Iteration 2, etc. (Figure 1).

Ethics

The study was approved by the ethics review board. Year 3 nursing students were recruited from a large, urban collaborative baccalaureate nursing program. Notices announcing the study were posted on the institution's learning management systems, and nursing students were e-mailed by the research assistant to indicate their willingness to be involved in the research. They were then sent the consent form and provided the dates and times for the study. When the students arrived at their assigned location, they read and signed the study consent before starting the study.

High-fidelity simulation intervention development

The investigators outlined the two simulation scenarios, each followed by a debriefing session. The two simulation conceptualizations were referred to as “Iteration 0.” Each of the simulations let nursing students encounter a different conflict management learning event with a simulated person acting as a senior nurse. Prior to their participation, nursing students received the following:

1) An online TeamSTEPPS™ (Team Strategies and Tools to Enhance Performance and Patient Safety) Mutual Support Microsoft® PowerPoint® (Microsoft® Corp., Redmond, WA, USA) and video (which demonstrated the use of a conflict resolution skill, the two challenge rule) [12],

2) A medication administration overview,

3) The patient’s diagnosis, medical history, medications, and treatment interventions.

Nursing students completed a dry-run of one of the two simulations (each followed by a debriefing session) and answered the following standard open-ended questions:

1) Which aspects of the simulation went well?

2) Which aspects of the simulation did not go well?

3) Were you able to carry out all the steps and procedures you had planned in the course of the medication administration process?

The investigator also posed a separate set of questions pertaining to the student’s behavior during the conflict management learning event in relation to the confederate:

1) How do you think you handled the conflict with the nurse?

2) What did it feel like to be a student nurse challenging a senior nurse?

3) Would you do anything differently in order to manage and/or resolve the conflict of a similar kind in the future?

Objective 1: Evaluation with the SDS and Appended Open-ended Question

After participating in the simulations and debriefing sessions, the nursing students completed the SDS. The SDS is a 20-item instrument that uses a 5-point measurement scale to evaluate a simulation scenario’s design features, as well as perceptions of the scenario’s objectives/information, problem-solving, feedback/guided reflection, and realism. The reliability of the SDS instrument using Cronbach’s alpha was 0.92 for the presence of simulation features and 0.96 for the perceived importance of features [13]. Student responses to the SDS were analyzed using descriptive statistics, means, medians, and standard deviations. The goal of the refinement process was not the setting of a specific baseline score for the simulations (e.g., an SDS score > 4.0).

In addition to completing the SDS (student version), nursing students were also asked to respond to the following open-ended inquiry: “The goal of this high fidelity simulation scenario is to teach a conflict resolution skill (the two-challenge rule). Bearing this goal in mind, please recommend a maximum of two changes, regardless of whether these changes relate to the design of the scenario, the conduct of the debriefing session, or both.” The principal investigator (PI) disregarded any recommendation recorded after the second recommended a change, without further consideration. Refinements to the scenario were restricted across iterations; more than two changes would complicate this difference attribution process. The open-ended question also helped the researchers gain a deeper understanding of possible beneficial modifications to the simulation scenarios and debriefing sessions based on the perspectives of the nursing students. Written responses to the open-ended question were content-analyzed for emerging codes and categories using conventional content analysis - a form of coding and categorizing data based on observation [14]. Two raters (the PI and another rater) operated independently and compared and evaluated the assignment of codes and categories. They then made the necessary changes before the next iteration.

The simulation’s refinement was based on data collected after each iteration. The two simulations (each followed by a debriefing session) were modified using the following algorithm for Day 1 (Iteration 1) of the refinement and decision rules (Figure 1). This process was repeated with final modifications to each of the simulations after the conclusion of the process. The results of all the nursing students' open-ended feedback were summarized and presented in tabular format.

Objective 2: Assessment of the Feasibility Outcomes of the Refinement Period for Each of the Simulations

The investigator evaluated the feasibility outcomes of each of the simulations refinement phases as follows: (a) maintained a record of the number of eligible nursing students invited to participate out of the total who agreed to participate in the study; (b) recorded the number of students who withdrew prior to or during participation (those who withdrew were contacted with a closed-ended question); (c) determined the time taken to complete the simulation scenarios and debriefing sessions (facilitators recorded completion times on a compliance data collection form). Times were compared across daily iterations of the refinement process. The final simulation iteration (on Day 5 of the process for each scenario) served as a baseline estimate of the time required to complete each scenario and its debriefing session. Moreover, the compliance data forms also indicated if facilitators pre-briefed students prior to participating in the simulation scenarios. 

## Results

Demographics

Half of the sample participated in refining the first simulation and the other half, the second simulation (each followed by a debriefing session). The demographic characteristics of the two groups were comparable. The mean age of participants was 21.6 years of age for the first simulation (SD 3, Mdn = 21, IQR =1) and 20.3 years of age for the second simulation (SD 0.5, Mdn = 20, IQR =1). The majority were female, held a high school diploma and spoke English at home.

Refinement of the first simulation scenario (followed by a debriefing session)

On Day 1, the SDS specific features index mean score was 3.45 (SD = 0.168) based on the evaluations from the first group of nursing students (n = 4). Two themes emerged that led to changes: the standardized person (SP), acting as the charge nurse could be more aggressive verbally by informing the student in a louder voice, “I am telling you to hang the intravenous (IV) bag, what’s the problem?” The second theme was that the students wanted the TeamSTEPPS PowerPoint to be converted into a landscape version from its original portrait format for easier viewing. The PI reviewed the changes with the confederates before Day 2.

The SDS specific features mean score for Day 2 of the refinement period was 3.588 (SD = 0.347). At the end of Day 2, the students recommended that the SP should appear more non-verbally aggressive by “crossing her arms.” The students also wanted a separate card with the two-challenge rule to refer to in the debriefing session. The SP was informed of the two changes that resulted in the third iteration of the simulation before engaging with the next day’s group of students.

At the conclusion of Day 3, the SDS specific features mean score was 4.087 (SD = 0.523). It was clear that the refinement process was improving the design features of the simulation. Nevertheless, there was still agreement as to what changes were required to further improve the simulation. The first of the two suggested changes was that they wanted to be advised that they could hang the intravenous (IV) bag with another team member, as they normally were assisted by their clinical instructor in the clinical domain (i.e., they wanted more clarity about their scope of practice in the scenario). Second, they requested that a case scenario in the pre-reading package be representative of a nursing-related information conflict because the one provided was too generic. These changes were implemented before Day 4 and were reviewed with the SP.

The SDS specific features mean score was 4.450 (SD = 0.091) on Day 4. Two themes resulted in changes being made to this iteration of the simulation scenario and debriefing session: students wanted a policy book to refer to with an IV administration guideline, and they wanted to be informed that they would have to react to a conflict in the scenario instead of only being told they would have to utilize the two-challenge rule. These changes were implemented before running the fifth version of the scenario and were reviewed with the SP.

On the final day, the SDS specific features mean score was 4.68 (SD = 0.144). This score represented the furthest move in the direction toward greater refinement from the score on Day 1. The two eligible identified themes that led to the changes implemented were that students wanted a more realistic medication administration record, and in the debriefing session, they wanted to be provided with other examples of conflict that may occur in the clinical domain. This final fifth version of simulation Scenario 1 and its debriefing session was the final version (see Table 1 for an overview of the changes).

**Table 1:** Simulation 1 - Categories Summarized in Tabular Format IV: intravenous; Sim Man: simulated manikin; TeamSTEPPS: Team Strategies and Tools to Enhance Performance and Patient Safety

Participants Thematic Agreements for Scenario Modifications Across Days - Scenario 1 Refinement			
Day 1			
Rank	# Agreements	Summarized Theme	Adopted Change
1	4	Actor should be more aggressive verbally	yes
2	3	TeamSTEPPS PowerPoint should be landscape format	yes
3	1	Request for a memory aid debrief	no
Day 2			
Rank	# Agreements	Summarized Theme	Adopted Change
1	4	Charge nurse should use more aggressive body language	yes
2	2	Wanted memory aid card and debrief	yes
3 (t)	1	Pre-learning pkg. should identify nursing conflict scenarios	no
3 (t)	1	Specify the scope of practice for students in the scenario	no
Day 3			
Rank	# Agreements	Summarized Theme	Adopted Change
1(t)	3	During briefing want to be advised they will hang the IV bag	yes
1(t)	3	Pre-learning pkg. should identify nursing conflict scenarios	yes
3(t)	1	Requested a policy book to refer to for skills	no
3(t)	1	Medication sheets should be in a separate binder	no
Day 4			
Rank	# Agreements	Summarized Theme	Adopted Change
1	3	Wanted to be informed of participation in conflict during the brief session	yes
2	2	Requested a policy book to refer too for skills	yes
3 (t)	1	Wanted to role-play the conflict scenario in the debriefing session	no
3 (t)	1	Medication sheets should be in a separate binder	no
3 (t)	1	Ensure equipment (i.e., a thermometer is readily available for the scenario)	no
Day 5			
Rank	# Agreements	Summarized Theme	Adopted Change
1 (t)	3	Wanted a more realistic medication administration record	yes
1 (t)	3	Wanted more examples of clinical conflict during the debrief	yes
3 (t)	1	Mr. Sim Man eyes closed	no
3 (t)	1	Role-play the conflict again in the debriefing session	no
Note. (t) denotes a rank tie between the number of agreements			

Refinement of the second simulation scenario (followed by a debriefing session)

The second simulation underwent the same refinement process as Scenario 1. On Day 1, the SDS specific features mean score was 3.738 (SD = 0.063) with two changes: the font in the pre-learning TeamSTEPPS PowerPoint was increased to 32 pt from 22 pt, and that the patient (Mr. Sim Man) creates more tension in the scenario by stating, “Do you know what you are doing?”

The SDS specific features mean score on Day 2, which increased slightly from Day 1, was 3.913 (SD = 0.103). Changes requested included the confederate appearing more demanding by raising his voice and students having access to a common clinical nursing skills book, such as Potter and Perry’s Canadian Fundamentals of Nursing (2013).

Day 3’s mean score increased to 4.075 (SD = 0.065). Nursing students requested that the intramuscular injection of vitamin B12, which was not familiar to them, be changed to iron intramuscular (IM) (as they had administered this in the clinical domain) and that the confederate should administer the IM injection regardless of whether the student challenged successfully.

Day 4 was 4.175 (SD = 0.155). The changes recommended for that day were (a) to have the SP put more verbal pressure on the student to “hurry up” and (b) to have the SP start challenging the student at the patient’s bedside and eventually to move away from the patient’s vicinity before a potential second challenge from the student.

The SDS specific features mean score of 4.750 (SD = 0.158) on the final Day 5 represented an increase of 1.01 from the mean score of 3.74 on Day 1. The first of two themes identified from student feedback was to separate the two-challenge rule on the pre-reading TeamSTEPPS PowerPoint, which would help students comprehend that the rule involved a two-step process. The second thematic suggestion was that more examples of information conflict should be provided in the debriefing session besides the one that occurred in the scenario. The changes implemented in response to the students’ feedback resulted in the final iteration of the second simulation scenario and its debriefing session (See Table 2 for an overview of the changes).

**Table 2:** Simulation 2 - Categories Summarized in Tabular Format ID: identification; IM: intramuscular; Sim Man: simulated manikin; TeamSTEPPS: Team Strategies and Tools to Enhance Performance and Patient Safety

Participants Thematic Agreement for Scenario Modifications Across Days - Scenario 2 Refinement			
Day 1			
Rank	# Agreements	Summarized Theme	Adopted Change
1 (t)	3	Increase TeamStepps pre-reading PowerPoint to 32 pt from 22 pt	yes
1 (t)	3	Mr. Sim Man should question "Have you given a needle before?"	yes
3 (t)	1	Confederate should be more verbally demanding with the student	no
3 (t)	1	Access to a policy book regarding IM injection	no
Day 2			
Rank	# Agreements	Summarized Theme	Adopted Change
1	3	Confederate should be more verbally demanding with the student	yes
2	2	Wanted access to a fundamental clinical skills textbook	yes
3 (t)	1	Change IM injection to one commonly administered in clinical setting	no
3 (t)	1	If student does challenge, allow to demonstrate the skill	no
3 (t)	1	Elaborate on other types of conflict in the debriefing session	no
Day 3			
Rank	# Agreements	Summarized Theme	Adopted Change
1	3	Confederate should be more verbally demanding with the student	yes
2	2	Change IM injection to iron IM versus vitamin B12	yes
3	1	Confederate should pressure the student to complete the IM	no
4	1	Two-challenge rule - a two-step process, separate the pre-reading	no
5	1	Elaborate on other types of conflict in the debriefing session	no
Day 4			
Rank	# Agreements	Summarized Theme	Adopted Change
1	3	Confederate moves away from the patient area if student challenges	yes
2	2	Confederate should pressure the student to hurry up	yes
3 (t)	1	Mr. Sim Man places pressure on the student; is impatient	no
3 (t)	1	If the student does challenge, allow to demonstrate the skill	no
3 (t)	1	Medication administration record, requires patient hospital ID	no
Day 4			
Rank	# Agreements	Summarized Theme	Adopted Change
1 (t)	2	Separate two-challenge rule in pre-reading package slides	yes
1 (t)	2	Provide more examples of conflict in the debriefing session	yes
3 (t)	1	If student does challenge, allow student to demonstrate IM mapping	no
3 (t)	1	Have a family member present	no
3 (t)	1	Teach an assertive communication skill in debriefing session	no
3 (t)	1	Students should go through the entire process of head-to-toe assessment	no
Note. (t) denotes a rank tie between the number of agreements			

In summary, 40 Year 3 nursing students participated in the refinement of two simulation scenarios (and their associated debriefing sessions). There was a clear and consistent improvement of the simulations, relative to the evaluative metric suggested by the SDS, that persisted across scenarios and days as the simulations were systematically refined.

The rate of participation of eligible Year 3 nursing students was 56% (45 out of 79). The five students who withdrew early represented an attrition rate of 1.12%. The students who withdrew were contacted with a follow-up closed-ended question used to determine their reasons for not completing the refinement process. Three students cited conflicts with their academic workloads; that is, they had numerous papers to write and had to study for the approaching examination week. The remaining two who withdrew said they had to work extra shifts in their part-time jobs.

The facilitators, who also conducted the debriefing sessions, observed the length of time it took each participating student to complete one of the two scenarios. There were 20 observations for the variable time (in minutes) for each simulation scenario over the separate one-week refinement periods. For simulation Scenario 1, each student participant spent a mean length of time of 15.1 minutes (SD = 3.3), median 15 minutes, ranging from 13.5 minutes to 18 minutes (interquartile range (IQR) = 4.5 minutes). During the refinement of Scenario 2, each student spent a mean length of time of 13.8 minutes (SD = 2.15), median 14.5 minutes, ranging from 12 minutes to 15 minutes (IQR = 6 minutes).

The facilitators also recorded the amount of time it took to complete each debriefing session that occurred after each of the simulation scenarios in the compliance data collection forms. On average, each student who participated in the debriefing following simulation Scenario 1 spent a mean length of time of 26.3 minutes (SD = 4), median 26.5 minutes, ranging from 25 minutes to 30 minutes (IQR = 5 minutes). For the debriefing session that followed Scenario 2, each participant spent, on average, a mean length of time of 28.9 minutes (SD = 3.4), median 30 minutes, ranging from 25 minutes to 32 minutes (IQR = 7 minutes).

## Discussion

Strengths

Educational simulations are being designed and implemented by faculty in nursing and health profession programs. At present, simulations do not typically undergo a systematic refinement process; rather, simulations may be evaluated by students using the SDS, and it is not clear if a "refinement" testing phase preceded the summative evaluation of the simulations’ design in the studies [4-6]. Additionally, the INACSL Simulation Design Standards include refinement criterion for simulations; however, descriptions were minimally described [15].

In other health professions disciplines, i.e., pediatric medicine, there is a call to test simulation scenarios to correct potential issues. Moreover, team members are encouraged to discuss their experiences and challenges by sharing videos of the simulation’s pilot runs with descriptions of lessons learned and how the scenarios should be managed [16]. Although sharing videos may be a helpful strategy, it lacks a systematic and iterative approach to track changes and generate the final version of the simulation intervention. Simulations may also undergo dry runs with faculty over a few hours, and changes are made on the fly, such as when nursing students provide feedback about errors of perception during the simulation. As a result, nursing students may not be getting a consistent educational experience from student-to-student or from group-to-group. The lack of a systematic refinement process for simulation interventions creates a greater risk of errors in simulation design going unacknowledged or uncorrected. What students experience in the context of simulation learning will likely remain in their minds and the risk of these errors being repeated in a clinical setting may also increase [17]. Therefore, applying a systematic refinement strategy to the simulations supports the best practices approach in pedagogical development and contributes to quality design.

Currently, no such strategy exists other than open-ended discussions or feedback provided by students through questionnaires, such as the SDS. However, there are challenges with the SDS, such as (1) when raters interpret its scores, the conceptual meaning they attribute to items is unknown and it is not clear which specific revisions in the simulation design features are required; (2) across studies that have used the SDS to evaluate simulations, there is minimal variability in ratings that reflect its discriminatory ability; (3) the conceptual analysis of items, such as fidelity and cueing in the SDS, have not been undertaken, thus establishing construct validity is problematic [18]. As a result of this novel refinement strategy (which includes the use of the SDS, an appended open-ended question, a refinement algorithm, and decision rules), a more holistic and programmatic approach to optimizing simulations may deal with some of the shortcomings of only using the SDS.

The development of quality simulations is important, given the findings that suggest an increase in active learning and confidence with students. The use of simulations in health profession education can encourage students to share their experiences, provides room for preconceptions/biases to be challenged constructively, allows students to make mistakes, and improves their nursing skills prior to going into the real world. Simulations also allow students to develop clinical competencies, promote teamwork, and improve care processes in a realistic and relatively safe environment without the potential of harm to patients [19-20]. Therefore, as educators, we have a moral duty to ensure high-quality simulation designs so that students can apply what has been learned in simulations accurately, transfer that knowledge to the real world, and engage in safe patient care.

This Phase 1 study is also a novel example of how the MRC framework can be applied to the design and testing of two simulations, each followed by a debriefing session [9-10]. Components of simulations may interact in unexpected ways; therefore, a systematic refinement approach is important to ensure that changes are made based on feedback in an explicit way. Developing, testing, implementing, and evaluating simulation interventions can be a lengthy process. However, all of the MRC phases are important, and neglecting adequate development and refinement work or not addressing the consideration of the practical issues of implementation will result in poorly designed simulations that may not meet learner outcomes.

Limitations 

There were several limitations to this study. The convenience sampling design did decrease the generalizability of the results. The sample was all female participants and English was their first language. These factors are important to consider since nursing programs typically include a diverse group of students and who may have prior post-secondary education and life experiences. Despite the pros of using a simulation refinement strategy as a means of enhancing quality and student learning, there are still disadvantages in its use. For example, the complexity of human life and experiences can never be fully replicated in a scenario-based simulation. Factors present in a clinical setting may not be evident in a simulation that takes place in a simulation center. Although the simulations have been refined in this study, it may be costly to optimize them due to faculty time constraints, a lack of faculty trained in simulation pedagogy, and support from leadership; moreover, it may be expensive to purchase and maintain needed software and hardware to run simulations [21]. Simulation models must also be manipulated by facilitators and simulation engineers in such a way to replicate a physiological response that may be desired under specific circumstances. Manipulating these systems in accordance with desired simulation goals might be cumbersome [19].

Lastly, we collaborated with 20 nursing students per simulation to provide feedback in order to optimize over five iterations, across five days. It will be important to further test this systematic refinement strategy under a variety of settings with fewer iterations and participants and observe the scores of the SDS, i.e., a three-iteration approach with nine participants. In usability studies, which assess product-related design problems and recommend changes, four or five participants allows one to discover 80% of a product’s usability issues and observing additional participants reveal fewer and fewer problems [22-23]. Perhaps, this refinement model can be tested with similar findings, as in the usability testing realm.

## Conclusions

The application of this systematic refinement strategy to each of the simulations was novel and improved the design elements despite the limitations of this study. The results also demonstrated that the changes made to the two simulations (each followed by a debriefing session) over two separate periods resulted in an increase of the SDS scores; thus, there was a trend in the evidence that the refinement strategy improved the overall quality of each of the simulations' design features. More studies are required that will replicate this strategy to assess the design elements of simulations and that will consider less than five iterations and fewer participants. The development and testing of simulation interventions is a time-intensive and collaborative process, but a refinement method is invaluable and allows replication by other educators and researchers to ensure quality design prior to implementing it into the curriculum and as interventions that evaluate the efficacy of simulation as a training methodology.

.
